# The relationship between small intestinal bacterial overgrowth and constipation in children – a comprehensive review

**DOI:** 10.3389/fcimb.2024.1431660

**Published:** 2024-06-27

**Authors:** Cristina Roxana Mares, Maria Oana Săsăran, Cristina Oana Mărginean

**Affiliations:** ^1^ Department of Pediatrics, “George Emil Palade” University of Medicine, Pharmacy, Sciences and Technology of Târgu Mures, Târgu Mures, Romania; ^2^ Department of Pediatrics 3, “George Emil Palade” University of Medicine, Pharmacy, Sciences and Technology of Târgu Mures, Târgu Mures, Romania; ^3^ Department of Pediatrics 1, “George Emil Palade” University of Medicine, Pharmacy, Sciences and Technology of Târgu Mures, Târgu Mures, Romania

**Keywords:** small intestinal bacterial overgrowth (SIBO), constipation, children, irritable bowel syndrome, functional gastrointestinal disorders

## Abstract

Small intestinal bacterial overgrowth (SIBO) is characterized by an increase in the bacterial population of the small intestine due to an imbalance between the amount of bacteria and the intestinal barrier. Pediatric SIBO presents with a wide spectrum of symptoms, ranging from mild gastrointestinal complaints to malabsorption or malnutrition. Breath tests are commonly used as noninvasive diagnostic tools for SIBO, but a standardized methodology is currently unavailable. Intestinal flora produces methane which slows intestinal transit and increases the contractile activity of small intestine. Emerging literature suggests a correlation between overgrowth of methanogenic bacteria in the intestines and constipation. Treatment of SIBO involves administration of antibacterial therapy in addition to management of underlying conditions and optimal dietary adjustments. However, research on antibiotic treatment for pediatric patients with constipation and SIBO is limited and has yielded conflicting results. In the current review, we summarize the state-of-the-art of the field and discuss previous treatment attempts and currently used regimens for SIBO patients with constipation, with a focus on pediatric populations.

## 1 Introduction

Small intestinal bacterial overgrowth (SIBO) consists of an increase in the bacterial content of the small intestine of more than 10^5^ colony-forming units (CFU)/mL (Bushyhead and Quigley, 2022; Skrzydło-Radomańska and Cukrowska, 2022), which produce gas in the small intestine, causing variable clinical aspects ranging from mild digestive symptoms (bloating, periumbilical pain) to more severe manifestations, such as malabsorption, malnutrition, nutritional deficiencies, as well as osmotic diarrhea (Hammer et al., 2022). SIBO can also determine irritable bowel syndrome (IBS) with symptoms of constipation-predominant syndrome in 54.6% of children and diarrhea-predominant type in the rest of children, according to Hutyra et al (Hutyra and Iwańczak, 2010).

The most common symptoms of SIBO are abdominal pain, diarrhea, constipation, flatulence, belching, foul-smelling stools with mucus, nausea and stunted growth (Cho et al., 2023). SIBO occurs when the balance between bacteria and the intestinal tract protection barrier is altered (Banaszak et al., 2023). Typically, the bacterial count in the proximal bowel is around 10^2^ CFU)/mL, which increases gradually towards the terminal ileum (Rana and Bhardwaj, 2008). The mechanisms that control bacterial proliferation are gastric acid secretion, digestive tract integrity, propulsive peristalsis and IgA immunoglobulins (Hammer et al., 2022). Therefore, numerous conditions in which these mechanisms are altered are associated with SIBO: ileo-cecal valve resection; small bowel diverticulosis; treatment with proton pump inhibitors, atrophic gastritis or gastric bypass which lower gastric pH; treatment with drugs that slow intestinal motility (antidiarrheals, anticholinergics) or abnormal small intestinal motility in different pathologies (celiac disease, inflammatory bowel disease, scleroderma, diabetes, Parkinson’s disease) (Sachdev and Pimentel, 2013; Marginean et al., 2017; Quigley et al., 2020; Hammer et al., 2022).

Additionally, an increase in lipopolysaccharide permeability exacerbates the inflammatory response causing chronic inflammation that can lead to SIBO. Inflammation of the small intestine in SIBO was demonstrated by elevated levels of pro-inflammatory cytokines (interleukin-1β -IL1β, interleukin 6 - IL6 and tumor necrosis factor α – TNFα) in the duodenal fluid (Rizos et al., 2022). Moreover, elevated levels of fecal calprotectin, a marker of intestinal inflammation have been reported in SIBO (Donowitz et al., 2016). Increase in ghrelin, leptin, or trimethylamine N-oxide (TMAO) levels, along with a higher gastric pH, could also contribute to the development of SIBO (Cheung and Wu, 2013; Augustyn et al., 2019; Banaszak et al., 2023).

In pediatric patients, the involvement of SIBO in various clinical conditions such as IBS (Chumpitazi et al., 2017), obesity (Esposito et al., 2020), failure to thrive (Collard et al., 2022) constipation, cystic fibrosis (Furnari et al., 2019) and short bowel syndrome has been investigated. Treatment with proton pumps inhibitors (PPI), altered gastrointestinal anatomy and living in impoverished conditions were identified as risk factors for SIBO in children (Leiby et al., 2010; Chumpitazi et al., 2017; Furnari et al., 2019; Esposito et al., 2020; Collard et al., 2022; Caporilli et al., 2023). SIBO prevalence varies between 14,3% in children with IBS (Korterink et al., 2015) and approximately 90% in children with failure to thrive (Collard et al., 2022) and chronic abdominal pain (Collins and Lin, 2011). However, data on the epidemiology of SIBO in children is limited by the small number of studies and varying test methodologies applied.

In the last 30 years, the Roma Foundation carried out the diagnostic framework and formulated the therapeutic recommendations for functional gastrointestinal disorders (Drossman, 2016). IBS, one of the main functional digestive disorders, has been described as a disturbance of the microbiota-gut-brain axis (Drossman, 2016). The main clinical features of IBS (abdominal pain, diarrhea, constipation) overlap those of SIBO, and several studies have shown a frequent association between the two entities (Drossman, 2016). Moreover, functional constipation, an entity with a high incidence in pediatric patients (van den Berg et al., 2006), has been linked with intestinal dysbiosis, related to an increase in the number of methane-producing intestinal bacteria (Leiby et al., 2010).

In this review, we *aimed* to investigate the link between SIBO and constipation in children. The article mainly addresses the constipation subtype of IBS and functional constipation.

## 2 Search strategy and selection criteria

A thorough literature search was conducted using PubMed, Scopus and Web of Science databases to gather all articles indexed until April 2024, investigating the association between SIBO and constipation in adults and children. Additionally, the “snowball” method was employed involving the examination of reference lists within articles, to identify additional pertinent studies (Wohlin et al., 2002).

Search terms included a combination of “SIBO”, “small intestinal bacterial overgrowth”, “small bowel bacterial overgrowth”, “intestinal methanogenic overgrowth (IMO)”, “ÏMO”, “methane”, “CH_4_”, “breath test”, “methane breath test”, “constipation”, “transit”, “motility”, “irritable bowel syndrome”, “irritable colon”, “child”, “pediatric”.

Two authors independently conducted an initial screen of titles and abstracts.

The *inclusion criteria* were population-based human studies, literature published in English and research articles examining the relationship between intestinal bacterial overgrowth and constipation. Full-text papers, including randomized controlled trials, prospective cohort studies, retrospective cross-sectional studies, and longitudinal studies, were included. *Exclusion criteria* comprised of studies which did not align with our research objectives, case reports, editorials, review articles, non-English publications, articles lacking free-available abstracts and duplicate entries. Additionally, abstracts and conference proceedings were omitted from the search results due to inadequate detail regarding the characteristics of the study population, diagnostic methodologies, or treatment modalities employed.

## 3 Results

### 3.1 Selection outcome

**Figure 1:**
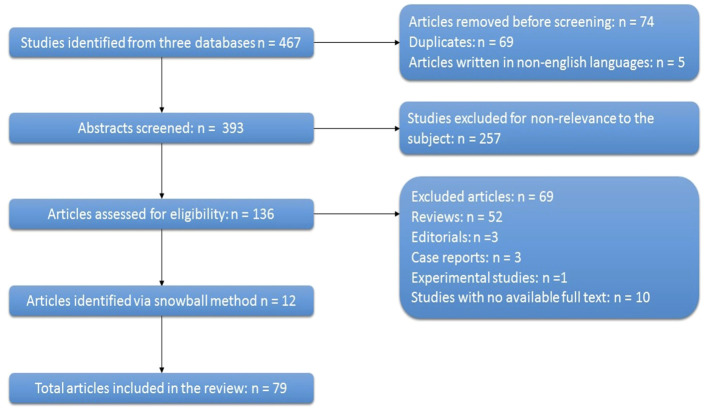
Flow diagram of studies assessed and included in the review. (n, number).

The database search yielded 467 articles. The 393 studies remaining after removing the duplicates and articles with full-text in other languages than English were screened by title or abstract. Only 136 of these studies were relevant to the research question out of which 69 were excluded for various reasons. By screening the reference list of the articles included we identified 12 additional studies. Therefore, we included in the review 79 articles which have complied with our inclusion and exclusion criteria, as summarized in **Figure 1**.

### 3.2 SIBO diagnosis

There have been numerous discussions regarding the interpretation of diagnostic tests for SIBO. In many studies, the gold standard for diagnosis was the presence of >10^5^ CFU/ml, determined from samples obtained by jejunal aspirate. However, in 2017, the North-American Consensus established the cutoff of ≥10^3^ CFU/mL as significant for the diagnosis of SIBO (Khoshini et al., 2008; Rezaie et al., 2017).

Jejunal aspiration is used for diagnosing SIBO, but is invasive and expensive, requiring a qualified gastroenterologist. In pediatric patients, the invasiveness of the procedure further restricts its use. Moreover, the sampling from the middle and distal regions of the small intestine is difficult, while sampling only from the proximal regions may cause false negative results. Also, the contamination of the samples with bacteria from the esophageal and oral flora can influence the culture results (Takakura and Pimentel, 2020). Given these disadvantages of the jejunal aspiration technique, breath tests (BTs) are frequently used to assess microbial overgrowth in the gut. These tests use different carbohydrate substrates, most commonly glucose and lactulose. The intestinal microflora transforms these substrates into hydrogen (H_2_) and methane (CH_4_), through anaerobic fermentation which are subsequently eliminated through respiration (Bond et al., 1971; Christl et al., 1992). The North American consensus defines a positive result for SIBO as an increase in H_2_ ≥ 20 parts per million (ppm) from baseline within 90 minutes of substrate ingestion and a CH_4_ level ≥ 10 ppm at any time of the test (Rezaie et al., 2017). The European guidelines for H_2_ and CH_4_ breath testing in adults and children (Hammer et al., 2022) published in 2021 refrain from defining a single cutoff for H_2_ and CH_4_ values in the diagnosis of SIBO, as the diagnostic criteria have not been sufficiently confirmed and uniformly accepted. The results of the breath test should be interpreted considering the pre-test probability of SIBO (the presence of risk factors or associated conditions, abdominal pain, bloating, malabsorption in the absence of another diagnosis on endoscopy and imaging) and serial tests with H_2_BT followed by a transit test with scintigraphy can be used in order to distinguish SIBO from rapid intestinal transit (Miller et al., 1997; Rao and Lele, 2002; Bratten et al., 2008; Yu et al., 2011; Hammer et al., 2022). Glucose is absorbed in the duodenum and jejunum, thus false negative BTs may occur (as high as 30–50%) if the bacteria are mainly located in the distal parts of the small intestine (Sellin and Hart, 1992; Saad and Chey, 2014). On the contrary, false positive results may be obtained in approximately 50% of patients with a rapid oro-cecal transit time (OCTT), as the glucose quickly reaches the colon (Lin and Massey, 2016). Lactulose is not absorbed in the small bowel and a BT using this substrate will show the contact with bacteria in the small bowel as well as in the colon (Saad and Chey, 2014). As a consequence, only early rises in the concentration of H_2_ during the lactulose BT indicate the presence of small bowel bacteria, although early increases may be secondary to a rapid OCTT (Yu et al., 2011). The specificity of both substrates in diagnosing SIBO is similar (80%-85%), but glucose BT is considered to have a higher sensitivity (62% versus 52%) (Hammer et al., 2022). For both substrates the diagnostic accuracy can be improved by combining a technique to evaluate OCTT (Zhao et al., 2010; Zhao et al., 2014; Lin and Massey, 2016). In the absence of scintigraphy to evaluate OCTT, glucose should be the preferred substrate, especially in non-surgical patients (Hammer et al., 2022). H_2_ detection through breath tests has been used since the 70s (Levitt and Donaldson, 1970), and more recently CH_4_ detection was introduced in the test protocols (Rezaie et al., 2017). Increase in H_2_ above the limit values during BTs have been associated with the diarrheal and the mixed form of IBS (Chen et al., 2018), while increases of CH_4_ have been linked to the constipation form of IBS (Hwang et al., 2010; Ghoshal et al., 2016). An acid pH in the colon, high dietary sulfate intake or increased methane production (as Archea use H_2_ to produce CH_4_) (Pimentel et al., 2020) can cause low rates of colonic H_2_ accumulation, resulting in false-negative results for hydrogen detection during BT. It has been suggested that measurement of breath CH_4_ concentrations may help in improving the sensitivity of BTs (Rezaie et al., 2017; Pimentel et al., 2020; Hammer et al., 2022).

Therefore, the limitations of BTs are related to false positive and negative results as well as the lack of a clear standardization of protocols. Also, the correct result of a breath test depends on the patient’s adherence to pre-test dietary and therapeutic restrictions, as well as a correct technique during the procedure. Despite these limitations, BTs remain valuable for SIBO diagnosis in pediatric patients, as they are practical and non-invasive. Notably, there haven’t been any significant side effects reported with the H_2_/CH_4_ BT, aside from occasional transient abdominal pain or vomiting during the procedure (Hammer et al., 2022). In pediatric patients, specific technical adjustments are implemented during BTs. For instance, in younger children, a face mask connected to a double bag via a T-valve is frequently employed. When a child cooperates both mentally and physically, adult breath collection techniques are utilized (Hammer et al., 2022). Other changes in BT protocol are the decrease in the minimum fasting period of 8 hours before the BT to 4–6 hours in infants. Moreover, glucose and lactulose substrate doses are calculated according to weight (Hammer et al., 2022).

### 3.3 SIBO and constipation

Functional gastrointestinal disorders (FGID) are commonly diagnosed conditions and are associated with transit abnormalities (Rao et al., 2011). Constipation, a common symptom in multiple FGIDs (Drossman, 2016) is caused by one of the following mechanisms: impaired rectal evacuation, IBS with constipation or secondary to slow transit caused by abnormalities of the enteric nerves (Pritchard et al., 2017). In pediatric patients, constipation is more commonly caused by changes in diet, toilet training or a painful defecation episode leading to withholding (Afzal et al., 2011; Colombo et al., 2015; Robin et al., 2018; Sharif et al., 2019).

IBS is one of the most commonly evaluated conditions linked to SIBO. It is defined as a functional gastrointestinal disorder, characterized by abdominal pain at least 4 days per month over at least 2 months, related to defecation or to changes in the form or frequency of stools. Importantly, the symptoms cannot be attributed to other medical conditions (Drossman, 2016).The main bowel symptoms determine the IBS-subgroups: IBS with constipation (IBS-C), IBS with diarrhea (IBS-D), and IBS with a mixture of constipation and diarrhea (IBS-M). This classification is important, as different subgroups require specific diagnostic tests and treatments (Menees et al., 2012). Prevalence of SIBO in IBS patients varies in different studies depending on the methodology and diagnostic criteria used (Reddymasu et al., 2010; Sachdeva et al., 2011; Ghoshal et al., 2020). A recent meta-analysis (Bratten et al., 2008) including 37 articles and 5379 IBS patients found a 36,7% global prevalence of SIBO in IBS patients, varying between 4,3% and 83,7%. This high variability of SIBO incidence can be explained by differences in the methodology of the included studies. SIBO was more prevalent in patients with IBS compared to controls, when assessed through the glucose hydrogen breath test and upper gut aspirate culture. However, the lactulose breath test (LBT) did not show a higher detection rate of SIBO in IBS patients compared to the control group. This finding suggests that LBT might lack specificity and could often yield false-positive results in healthy individuals. Patients with IBS-D were more likely to have SIBO than patients with other subtypes of IBS (Efremova et al., 2023).

A similar global SIBO prevalence in patients with IBS (31%) was obtained in another meta-analysis that included 25 studies (Shah et al., 2020). SIBO prevalence in IBS patients was 35,5% using BT and only 13.9% using cultures from aspirates. Similar to the previous meta-analysis, LBT led to a much higher prevalence of SIBO in IBS, compared to glucose breath test (GBT) and cultures. Furthermore, SIBO prevalence was greater in patients with IBS-D (35.5%) compared with patients with IBS-C (22.5%) and IBS-M (25.2%).

Studies in children have found a prevalence of SIBO in IBS ranging from 14,3% to 91%. The high variability may be due to different inclusion criteria and diagnostic methodologies. Studies which used glucose as a substrate found a lower prevalence [14,3% (Korterink et al., 2015) – 34% (de Boissieu et al., 1996)], while lactulose yielded a higher prevalence of the same condition [39% (Ojetti et al., 2014) – 91% (Collins and Lin, 2011)].

A large meta-analysis from 2020 (Shah et al., 2020) included 3192 patients with IBS and 3320 controls and found that patients with IBS-C had a three-times higher prevalence of methane positive SIBO (25.3%) compared with patients with IBS-D (8.8%). The OR for methane-positive SIBO in patients with IBS compared with controls was 1.2. SIBO was much more prevalent in patients with IBS versus controls. Moreover, the prevalence of SIBO diagnosed through LBT was higher than the one established though GBT in both patients and controls (Shah et al., 2020). Similarly, in a meta-analysis conducted by Kunkel et al, a significant association was found between methane detected on breath tests and constipation (OR = 3.51) (Kunkel et al., 2011).

However, other studies did not find a correlation between SIBO and diarrhea or constipation, nor between the prevalence of methane-positive SIBO in chronic constipation compared to controls (Reddymasu et al., 2010).

Another meta-analysis revealed that the incidence of methane-positive SIBO in patients with IBS was 25%, which was not substantially different from the control group (Chuah et al., 2022). Nevertheless, methane-positive SIBO was more prevalent in the constipation subtype compared to IBS-D (OR = 3.1). LBT yielded positive results for methane-positive SIBO nearly three times as often as GBT (29.0% vs 11.5%) (Chuah et al., 2022).

Different tests have been used to determine the impact of SIBO on transit times in the small intestine and colon. Suri et al (Suri et al., 2018) used scintigraphy to study SBT (small bowel transit) and CT (colonic transit) in patients with hydrogen-positive (H-SIBO) and methane-positive (M-SIBO) LBT, and found that the presence of SIBO does not affect SBT nor CT. However, M-SIBO exhibited significantly delayed SBT and CT compared to H-SIBO, indicating the presence of delayed motility in patients with elevated methane levels, as found on LBT (Suri et al., 2018). On the contrary, another study (Yu et al., 2011), which employed oro-caecal scintigraphy and LBT in IBS patients, concluded that abnormal increases in hydrogen levels measured during the breath test could be attributed to variations in oro-caecal transit time rather than SIBO. However, it’s worth noting that this study did not include measurements of breath methane.

A study utilizing a wireless motility device compared intestinal transit patterns and breath tests among individuals with IBS and discovered no discernible link between SBT and abnormal breath H_2_ or CH_4_ excretion (DuPont et al., 2014). The study also showed that 76% of IBS patients exhibited prolonged gastric emptying times, with IBS-C being associated with increased gut transit times (DuPont et al., 2014).

Lastly, a study using a barostat (Grover et al., 2008) has shown that methane-producing IBS patients have higher urge thresholds and higher baseline levels of colon phasic contractions than SIBO-negative IBS patients, and report an increased consistency of stools.

CH_4_, a product of intestinal fermentation, has been shown to directly slow intestinal transit and cause constipation in animal models, as well as humans (Takakura and Pimentel, 2020). Multiple studies have demonstrated an association between positive methane breath test and constipation, as well as between the degree of constipation and breath CH_4_ levels in subjects with IBS (Chatterjee et al., 2007; Attaluri et al., 2010; Furnari et al., 2012). Increased methane production was also found in diverticulosis, a condition frequently associated with constipation (Weaver et al., 1986; Yazici et al., 2016). CH_4_ appears to amplify neuronal activity in the intestine through the anticholinergic pathway and initiates slowing of peristalsis in the proximal intestinal segment. Contractile activity in the proximal intestinal segments is inhibited through a feedback loop when the distal segments are exposed to excess amounts of methane. Another proposed mechanism is the generation of non-propagating small bowel contractions, leading to delayed transit times (Pimentel et al., 2003b; Park et al., 2017; Suri et al., 2018). In another study methane- producing IBS patients had lower postprandial serotonin levels compared to the hydrogen-producing group. As serotonin is a key mediator of the peristaltic reflex, it may be a cause of delayed intestinal peristalsis in methane-producing patients (Pimentel et al., 2004). *Methanobrevibacter smithii* (*M. smithii*), a member of the Archaea domain, has been linked to constipation-predominant IBS and is the main methanogen responsible of CH_4_ production. Because archaea are not bacteria, intestinal methanogenic overgrowth (IMO) and not SIBO is a more appropriate term for *M. smithii* overgrowth (Cho et al., 2023).

### 3.4 SIBO and constipation in children

In children few studies have investigated the association between SIBO and constipation. Ojetti et al. investigated 18 children with myelomeningocele, a condition frequently associated with constipation, and diagnosed SIBO in 38% of the patients, using LBT (Ojetti et al., 2014). Interestingly, all children who produced CH_4_ showed a delayed OCTT with a lower frequency of evacuation.

Similarly, a study using LBT in children with fecal retentive incontinence found a prevalence of SIBO of 42% (Leiby et al., 2010). Moreover, 48% of patients with fecal incontinence showed high CH_4_ values compared to only 10% in the control group. Fecal impaction scores were significantly increased in children with encopresis who were methane producers.

Soares et al., investigated the relationship between CT time, determined by radio-opaque markers and CH4 production in children with constipation (Soares et al., 2005). An increase in CH_4_ production was found in 73.5% of children with constipation and incontinence, but only in 16.7% of children with constipation and no incontinence. Similarly, another study found increased CH_4_ production in 65% of encopretic patients and in only 11% of patients with constipation and no encopresis (Fiedorek et al., 1990). Therefore, pediatric constipated patients with encopresis are more likely to be CH_4_ producers than constipated patients without encopresis (Soares et al., 2005). Soares et al. also found that CH_4_ producers had a prolonged CTT, which decreased after successful treatment (Soares et al., 2005). Similar results indicated that in children with IBS, CH_4_ production correlated positively with whole intestinal transit time and negatively with bowel movement frequency (Chumpitazi et al., 2017).

Other studies in children have failed to demonstrate significant correlations between breath tests and transit changes. Scarpellini et al. used LBT to measure H_2_ and CH_4_ in 43 children with IBS and 56 controls (Scarpellini et al., 2009). They observed a higher prevalence of abnormal LBT results among patients diagnosed with IBS (65%, 28 out of 43 patients) compared to controls (7%, 4 out of 56 patients). However, no association between CH_4_ production and intestinal transit changes was found (Scarpellini et al., 2009). A similar result was obtained in a study on 54 children with IBS, which showed no strong correlation between symptoms (constipation, diarrhea, bloating, abdominal pain, nausea) and H_2_ and CH_4_ breath test results (Peinado Fabregat et al., 2022). However, there was a small correlation between the presence of diarrhea and nausea and increased H_2_ production (Peinado Fabregat et al., 2022). Contrarily, Hutyra et al. found a higher prevalence of SIBO in children with constipation-predominant IBS (54.55%) compared to diarrhea-predominant IBS (2.86%) (Hutyra et al., 2009).

Mello et al. studied the association between CH_4_ production and SIBO in two socioeconomically distinct categories of children in Brazil (Mello et al., 2012). One group consisted of children living in poor conditions in a slum, while the second group of children came from socioeconomically advantaged families. The study revealed a high CH_4_ production, regardless of SIBO presence in children living in unfavorable environments. However, there wasn’t a clear correlation between SIBO and increased CH_4_ production (Mello et al., 2012). Among children residing in slum areas, there was no obvious link between CH_4_ production and constipation. Conversely, within the private school group, 3 out of 8 children who produced CH_4_ complained of constipation (Mello et al., 2012).

SIBO presence was also studied in pediatric patients with Abdominal Pain–Related Functional Gastrointestinal Disorders (AP-FGID). Korterink et al. found that 14.3% of AP-FGID patients were diagnosed with SIBO, and IBS was significantly more frequent in children with SIBO compared to those without SIBO (Korterink et al., 2015). A similar prevalence of SIBO (20.6%) was identified in another study on 68 children with AP-FGID (Lee et al., 2022). Loose stools were notably more prevalent among patients testing positive for H_2_ or CH_4_, although no other correlations with bowel symptoms were identified.

**Figure 2:**
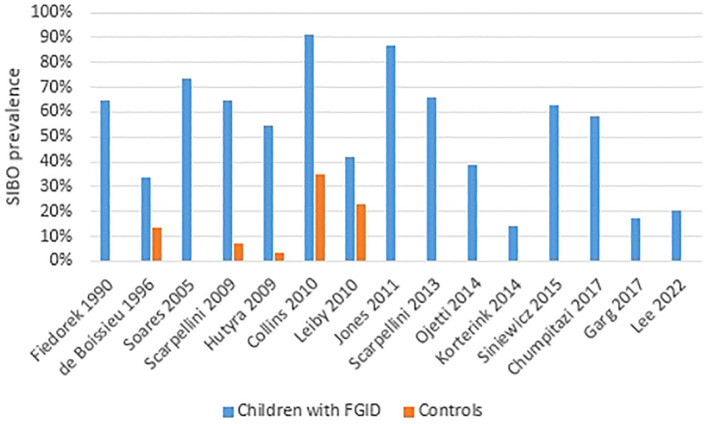
SIBO prevalence in children with functional gastro-intestinal disorders. The majority of studies did not include a control group.

**Table 1** shows the main characteristics and results of the studies published in pediatric patients with FGID. The prevalence of SIBO in studies including children with FGID is summarized in **Figure 2**.

**Table 1:** SIBO and functional gastrointestinal disorders in children.

Authors	Year	Study population - children	Diagnostic test	Results
de Boissieu et al (de Boissieu et al., 1996)	1996	50 children with chronic diarrhea, abdominal pain, or both, further included into 4 groups: - Group 1 – subjects with positive BT treated with antibiotics - Group 2 – subjects with negative BT - Group 3 – controls - Group 4 – patients with bacteriologically proven SIBO	Glucose H_2_ BT Positivity was defined by a change in H_2_ value ≥ 10 ppm after ingestion of glucose.	34% SIBO prevalence
Soares et al (Soares et al., 2005)	2005	- 40 children with chronic constipation evaluated before and after 6 weeks of treatment	CH_4_ BT Positivity was defined as a methane concentration > 3 ppm	73,5% SIBO prevalence in patients with constipation and encopresis and 16,7% in patients with isolated constipation. CH_4_-positive patients had a prolonged colonic transit time
Scarpellini et al (Scarpellini et al., 2009)	2009	- 43 children with IBS (Rome II criteria) and - 56 healthy controls	Lactulose H_2_/CH_4_ BT. Positivity was defined as an early rise in H_2_ or CH_4_ excretion of > 20 ppm within the first 90 min.	SIBO prevalence: Cases: 65%/Controls: 7% No correlation between H_2_/CH_4_ values and bowel habits
Hutyra et al (Hutyra et al., 2009)	2009	- 136 children with functional dyspepsia, chronic abdominal pain and IBS; - 28 controls, children treated for other pathologies	Lactulose H_2_ BT Positivity was defined as an increase in H_2_ > 20 ppm within the first hour	54.55% SIBO prevalence in constipation predominant IBS, 2.86% in diarrhea predominant IBS
Collins et al (Collins and Lin, 2011)	2010	- 75 Children with chronic abdominal pain randomized into two groups, one treated with Rifaximin and one with placebo; - 40 healthy controls	Lactulose H_2_ BT Positivity was defined as a rise in H_2_ >20 ppm in the first 90 min	SIBO prevalence of 91% in cases and 35% in controls Abnormal LBTs persisted after treatment in 80% children who received Rifaximin and 86% children who received placebo
Leiby et al (Leiby et al., 2010)	2010	- 50 children with fecal incontinence; - 39 controls with gastrointestinal symptoms but without fecal incontinence	Lactulose H_2_/CH_4_ BT Positivity was defined as an increase in H_2_ > 20 ppm or in CH_4_ >10 ppm over baseline at < 60 min. Patients were considered CH_4_ producers if their level was >3 ppm at any point in the study	SIBO prevalence 42% in cases, 23% in controls
Jones et al (Jones et al., 2011)	2011	287 children with chronic diarrhea, abdominal pain, bloating or irritability	H_2_ and CH_4_ levels Positivity was defined as an increase in H_2_ >10 ppm over baseline in the initial 45 min of the test. Patients were classified as H_2_ or CH_4_ producers if they produced >10 ppm of these gases at any time point	87% SIBO prevalence
Scarpellini et al (Scarpellini et al., 2009)	2013	50 children with IBS (Rome II criteria) treated with Rifaximin	Lactulose H_2_/CH_4_ BT Positivity was defined as an increase in H_2_ or CH_4_ excretion >20 ppm within the first 90 min	66% SIBO prevalence 64% LBT normalization rate after treatment
Ojetti et al (Ojetti et al., 2014)	2013	18 children with myelomeningocele and constipation	Lactulose H_2_/CH_4_ breath test. Positivity was defined as an increase in H2 or CH4 excretion >20 ppm within the first 90 min	39% SIBO prevalence
Korterink et al (Korterink et al., 2015)	2014	161 children with abdominal pain related functional gastro-intestinal disorders (Rome III criteria), divided into two groups: SIBO-positive and SIBO-negative	GHBT. Positivity was defined as fasting breath H_2_ concentration > 20 ppm or increase in H_2_ >12 ppm over baseline value	14.3% SIBO prevalence
Siniewicz-Luzenczyk et al (Siniewicz-Luzeńczyk et al., 2015)	2015	100 children with abdominal pain	Positivity was defined by a baseline concentration of H_2_ > 20 ppm or an increase in H_2_ > 20 ppm in the first hour of the test.	63% SIBO prevalence 88% HBT normalization rate after Rifaximin treatment and improvement in symptoms
Chumpitazi et al (Chumpitazi et al., 2017)	2017	87 children with IBS (Rome III criteria)	Subjects who excreted ≥ 3 ppm of CH_4_ in at least one of the breath samples were characterized as CH_4_ producers	LBT CH4 correlated positively with whole intestinal transit time and negatively with bowel movement frequency. 58.6% were CH_4_ producers No differences by IBS subtype in H2 or CH_4_ production
Garg et al (Garg et al., 2017)	2017	- 62 children with chronic abdominal pain underwent BT - 21 were diagnosed as lactose intolerant and 8 as SIBO-positive	GHBT and LHBT SIBO positivity defined as an increase in H2 < 10 ppm in 30 minutes Lactose intolerance defined as an increase in H2 > 20 ppm	17% SIBO prevalence on LHBT
Lee et al (Lee et al., 2022)	2022	68 children with functional abdominal pain disorders (Rome IV)	Glucose H_2_ and CH_4_ breath test Positivity was defined by an increase in H_2_ > 12 ppm above baseline within 90 minutes or an increase in CH_4_ > 10 ppm above baseline within 90 minutes	20.6% SIBO prevalence

BT, Breath test; LBT, lactose breath test; LHBT, lactose hydrogen breath test; GHBT, glucose hydrogen breath test; H_2_, Hydrogen; CH_4_, Methane; SIBO, Small intestinal bacterial overgrowth; IBS, irritable bowel syndrome; ppm, parts per million.

### 3.5 SIBO treatment in adults and children

Treating SIBO in both children and adults involves a complex approach aimed to reduce bacterial overgrowth, relieve symptoms and address any underlying causes. Treatment strategies often involve a combination of antibiotics, dietary modifications and probiotics (Quigley et al., 2020). Antibiotics are the first line of treatment for SIBO, and are often empirically initiated, due to difficulties in obtaining culture aspirates and isolation of bacterial pathogens. Commonly prescribed antibiotics for SIBO include rifaximin, neomycin and metronidazole (Collins and Lin, 2011; Cho et al., 2023).

Neomycin is one of the first antibiotics studied in adult IBS patients. Although it was effective, resulting in a 35% improvement in IBS symptoms composite scores compared to 11% for placebo, its use was limited by the numerous side effects (Pimentel et al., 2003a). Rifaximin, the most studied antibiotic in the treatment of IBS, inhibits bacterial RNA synthesis, thereby disrupting the growth and reproduction of bacteria in the gut (Scarpignato and Pelosini, 2005; Koo et al., 2012). Unlike many other antibiotics absorbed systemically into the bloodstream, the effects of rifaximin are mainly restricted to the gastrointestinal tract after oral administration (Scarpignato and Pelosini, 2005). Because of its safety features (less frequently reported systemic side effects commonly associated with other antibiotics), rifaximin has been approved by the FDA for the treatment of IBS-D (Pimentel and Lembo, 2020). Rifaximin has been shown to eradicate bacterial overgrowth in up to 80% of adult patients diagnosed with SIBO (Scarpellini et al., 2013). A meta-analysis (Gatta and Scarpignato, 2017) that included 32 studies and 1331 adults SIBO patients identified an overall eradication rate of 70% of bacterial overgrowth, with adverse effects occurring in less than 5% of cases. A dose-dependent effect was demonstrated, the most commonly used dose being 1200 mg per day. In addition, rifaximin is more effective in reducing symptoms in IBS patients compared to placebo (Menees et al., 2012). Notably, symptom improvement was seen more frequently in patients treated with rifaximin compared to other antibiotics, such as neomycin, doxycycline, amoxicillin/clavulanate and ciprofloxacin (Yang et al., 2008).

In adult patients with IBS-C, treatment with specific antibiotics decreased CH_4_ levels, which correlated with relief of constipation (Pimentel et al., 2006; Low et al., 2010). Both neomycin and rifaximin have been shown to reduce constipation in IBS-C, but using a combination of the two drugs appears to be more effective (Low et al., 2010). Additionally, reduction of CH_4_ to undetectable levels (< 3 ppm) on repeat BT was obtained in 33% of patients treated with neomycin, 28% patients treated with rifaximin and 87% patients treated with both antibiotics (Pimentel et al., 2014).

**Figure 3:**
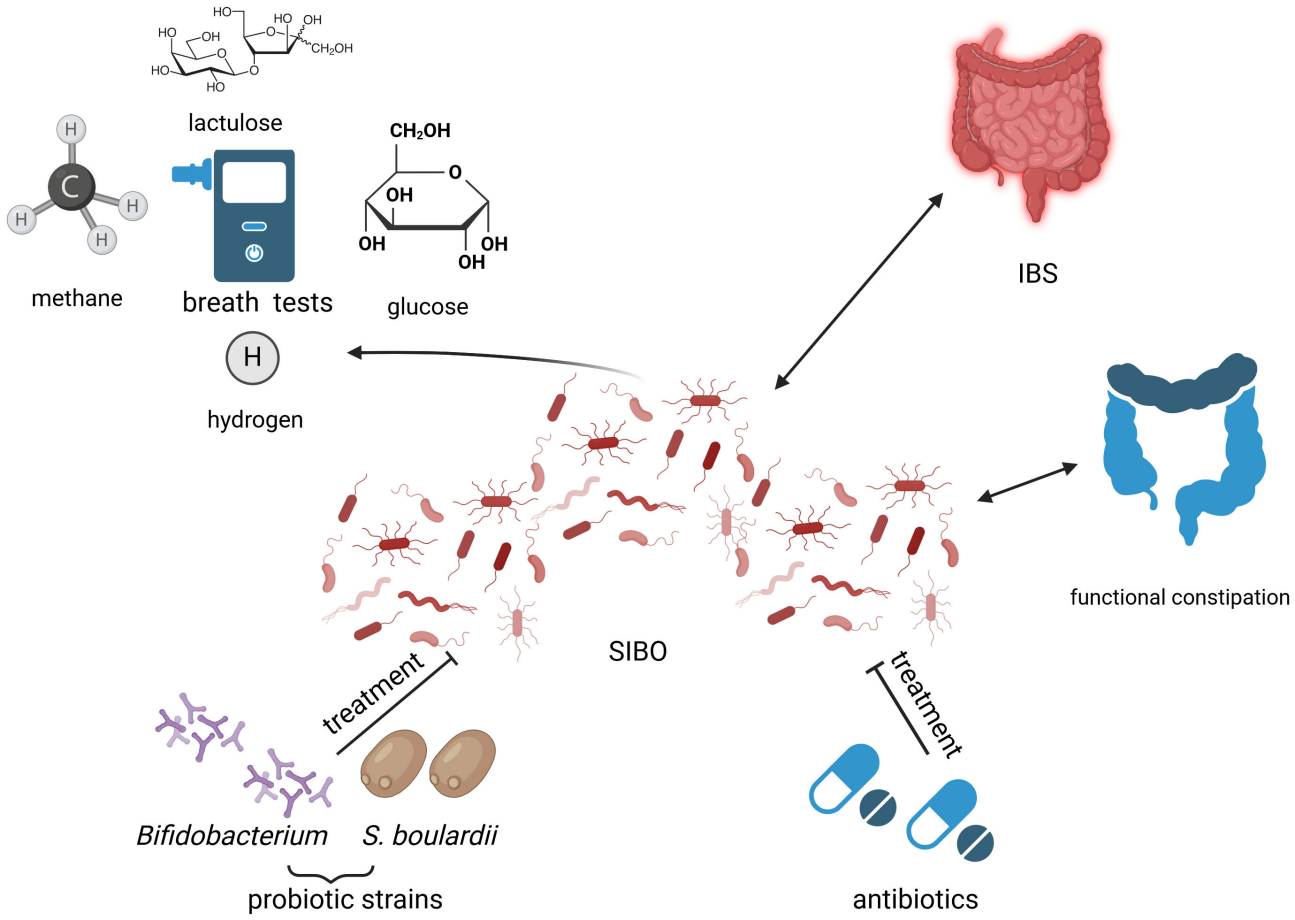
Small intestinal bacterial overgrowth (SIBO) in relation to childhood constipation: diagnostic and treatment opportunities. Created with BioRender.com (https://biorender.com/) Small intestinal bacterial overgrowth (SIBO) has been linked to functional constipation and irritable bowel syndrome (IBS). The possibility of SIBO depiction through multiple types of breath tests (BTs), such as lactulose BT, hydrogen BT, glucose BT or methane BT leads to miscellaneous results of currently available studies, due to methodology-related disparities. Current treatment options of SIBO include probiotics belonging to the Bifidobacterium genre and *Saccharomyces boulardii* (*S. boulardii*), as well as antibiotics, but research on this matter is ongoing. IBS, irritable bowel syndrome; *S. boulardii, Saccharomyces boulardii*; SIBO, small intestinal bacterial overgrowth.

In the pediatric population, data are limited regarding the use of antibiotics to treat SIBO. The effect of rifaximin in children with IBS was evaluated in a study that included 33 subjects with a positive LBT (Scarpellini et al., 2013). The breath test normalized in 64% of cases treated with rifaximin 200 mg daily for 7 days. Furthermore, visual analogic scale scores for gastrointestinal symptoms improved after successful treatment. Similar results were reported by Siniewicz-Luzenczyk et al, with a normalization of BT in 88% cases after treatment (Siniewicz-Luzeńczyk et al., 2015). Another course of antibiotics was used to treat children with SIBO consisting of the combination of trimethoprim-sulphametoxazole 30 mg/kg daily and metronidazole 20 mg/kg daily for 14 days, which normalized BTs in 95% of cases (Tahan et al., 2013). Furthermore, different antibiotics and probiotics regimens have been studied in children with SIBO and a positive GBT or LBT (Peinado Fabregat et al., 2022). Treatment with the combination of probiotics and antibiotics demonstrated a better resolution of symptoms compared to treatment with antibiotics alone (81% vs. 67.7%). The effects were similar with respect to the antibiotic used. An overview of the current therapeutic options of SIBO has been illustrated in **Figure 3**.

Non-pharmacological methods were also used in the treatment of SIBO. Dietary approaches for managing SIBO typically involve reducing the intake of fermentable substances such as fiber, sugar alcohols and sweeteners like sucralose (Staudacher and Whelan, 2017; Souza et al., 2022). These strategies are often based on dietary guidelines for IBS, emphasizing low-FODMAP diets, which restrict fermentable oligosaccharides, disaccharides, monosaccharides, and polyols. However, the mechanisms behind clinical improvements resulting from dietary changes remains unclear. It is uncertain whether these changes primarily affect the intestinal microbiota or simply reduce fermentation and gas production.

The role of probiotics in the treatment of SIBO has also been investigated. A recent meta-analysis has found that probiotics appeared to reduce hydrogen production (Zhong et al., 2017). In randomized clinical trials examining probiotic use in SIBO, variations were observed in the strains used and the duration of treatment (Souza et al., 2022). One study investigated the effect of *Bifidobacterium* in 126 patients diagnosed with gastrointestinal cancer and SIBO (Liang et al., 2016). Following the treatment regimen, SIBO was eradicated in 81% of individuals administered probiotics, compared to 25.4% in the placebo group. Furthermore, symptoms were significantly reduced in the probiotic group but not in the placebo group.

Similar results were reported in a study on IBS- D and SIBO (Bustos Fernández et al., 2023). Participants received either *Saccharomyces boulardii CNCM I-74* (Sb) along with dietary advice (DA) or DA alone. The researchers observed a more pronounced reduction in hydrogen excretion in the Sb group compared to the DA group. Additionally, Sb supplementation led to an improvement in digestive symptoms.

Conversely, in a randomized, double-blind trial (Stotzer et al., 1996) involving 17 individuals diagnosed with SIBO, *Lactobacillus fermentum KLD* did not produce significant changes in BT outcomes, clinical symptoms, or stool frequency when compared to the baseline measures.

In children with SIBO we identified only one retrospective report studying the effect of probiotics in a limited group of only 10 patients (Ockeloen and Deckers-Kocken, 2012). Of these, 7 children had an improvement in their abdominal complaints after treatment with *Bifidobacterium* and *Lactobacillus*, but the difference was not statistically significant.

Additionally, most studies exhibited moderate methodological quality, therefore no recommendations for standardized treatment are currently available (Pimentel and Lembo, 2020; Souza et al., 2022).

## 4 Future research directions and limitations of current data

To the best of our knowledge, this review is the first to explore the association between SIBO and constipation in children. A strong point of this review was the comprehensive literature search of all the studies including pediatric patients with SIBO and constipation.

In the adult population, there are numerous studies investigating the diagnosis and treatment of SIBO. However, in children research related to this topic is very limited, even less regarding the SIBO-constipation relationship. Technical difficulties in performing both digestive endoscopy with the collection of jejunal aspirate, and respiratory tests in children largely explain this paucity of studies.

The main limitations of our review are generated by the small number of studies available. The majority of these studies featured small sample sizes and lacked control groups. In addition, we noticed a considerable variability between studies regarding diagnostic methods and threshold values for BT, therefore strong conclusions could not be formulated. Most studies have used lactulose as a substrate for BT, although the most recent European consensus recommends the use of glucose because it leads to a higher sensitivity (Hammer et al., 2022). **Figure 2** also highlights the miscellaneous methods used for breath test related diagnosis of SIBO.

Cut-off points for SIBO diagnosis using BT have not been adapted to the pediatric population. This is problematic since recent studies have demonstrated variations between the gut microbiota of children and adolescents compared to adults (Hollister et al., 2015; Derrien et al, 2019).

In adult studies, a positive CH_4_ BT has been associated with constipation and delayed intestinal motility (Chatterjee et al., 2007; Attaluri et al., 2010; Furnari et al., 2012; Suri et al., 2018). Nevertheless, research on children has produced inconsistent results on this issue. Several studies have suggested that children experiencing constipation and encopresis exhibit elevated CH_4_ production and a delayed OCTT (Fiedorek et al., 1990; Soares et al., 2005; Ojetti et al., 2014). Conversely, other studies found no correlation between BT results and bowel movement frequency in children with isolated constipation (Scarpellini et al., 2009; Peinado Fabregat et al., 2022).

Lastly, there is also a paucity of studies focusing on the treatment of SIBO in children. While rifaximin has similar positive outcomes in children as in adults (Scarpellini et al., 2013; Siniewicz-Luzeńczyk et al., 2015), a previous study noted a higher rate of SIBO eradication with trimethoprim-sulphamethoxazole (Tahan et al., 2013), indicating that additional studies are necessary.

## 5 Conclusions

SIBO is a poorly understood condition with variable clinical manifestations, ranging from mild symptoms to malabsorption and failure to thrive. Identifying SIBO in pediatric patients is crucial to prevent long-term complications and optimizing growth and development.

In adults, numerous studies have demonstrated a significant link between intestinal methanogenesis and constipation. However, as this review points out, research on this correlation in the pediatric population has yielded conflicting results, potentially due to the limited sample size and methodological variations across studies.

Additionally, determining SIBO incidence in pediatric patients remains challenging due to the lack of standardized diagnostic criteria and limited studies focusing on this age group. Rigorous case-control studies on large population samples, using glucose as a substrate and simultaneously measuring intestinal transit time via scintigraphy or other diagnostic methods, could improve the diagnostic criteria for SIBO, as currently suggested through European consensus. Further investigations are necessary to establish a universally accepted diagnostic criteria and threshold values for pediatric SIBO and these values are difficult to establish without future research enrolling control groups, missing from most available studies. Moreover, exploring the impact of diet, probiotic therapy or the adaptation of effective antibiotics from adult treatments in pediatric patients should be addressed in future studies, as this subject is worth exploring from a therapeutic point of view in children as well.

## Author contributions

CRM: Formal analysis, Investigation, Methodology, Writing – original draft, Writing – review & editing. MS: Conceptualization, Investigation, Methodology, Project administration, Resources, Supervision, Validation, Visualization, Writing – original draft, Writing – review & editing. CM: Formal analysis, Investigation, Methodology, Writing – original draft, Writing – review & editing, Conceptualization, Data curation, Project administration, Validation, Visualization.
